# A Social Cognitive Theory Approach to Understanding Parental Attitudes and Intentions to Vaccinate Children during the COVID-19 Pandemic

**DOI:** 10.3390/vaccines10111876

**Published:** 2022-11-07

**Authors:** Ying Zhu, Michael Beam, Yue Ming, Nichole Egbert, Tara C. Smith

**Affiliations:** 1 College of Communication and Information, Kent State University, 318 University Library, 1125 Risman Drive, Kent, OH 44242, USA; 2 School of Emerging Media & Technology, Kent State University, 550 Hilltop Drive, Kent, OH 44242, USA; 3 School of Communication Studies, Kent State University, P.O. Box 5190, Kent, OH 44242, USA; 4 College of Public Health, Kent State University, 800 Hilltop Drive, Kent, OH 44242, USA

**Keywords:** COVID-19, child vaccination, social cognitive theory, political polarization, survey methods

## Abstract

The distribution of the COVID-19 vaccine represents a path towards global health after a worldwide pandemic. Yet, the U.S. response to the vaccination rollout has been politically polarized. The aim of this paper is to contribute to the understanding of the contextual factors that influence parents’ attitudes towards health officials and their intention to vaccinate children, focusing on communication behaviors, personal factors, and geographic locations. We use Bandura’s triadic reciprocal determinism (TRD) model which posits reciprocal influence between personal factors, environmental factors, and behaviors. We found that personal factors (having younger children and identifying as Republican partisans), and the behavioral factor of conservative news use were significantly related to more negative attitudes towards health officials and lower vaccination intentions. Conversely, Democrats and liberal news use were significantly related to warmer attitudes and greater vaccination intentions. The environmental factor of geographic location across four states with different partisan dynamics was not significantly related to attitudes and behavioral intentions. Results from a post-hoc analysis show that news media use and partisanship were the strongest correlates of parents’ attitudes towards health officials. This evidence points to the politicization of the COVID-19 vaccine being a key consideration regarding vaccine uptake.

## 1. Introduction

For years, researchers and public health officials have voiced growing concerns about factors that negatively influence parents’ uptake of child vaccinations such as the politicization and misinformation surrounding vaccines [1,2]. The development of successful COVID-19 vaccines has brought new widespread attention to the importance of parents’ attitudes towards vaccinating children. Improving child vaccination rates is a crucial factor in protecting against the direct and indirect dangers of COVID-19. This study employs the triadic reciprocal determinism model (TRD) from social cognitive theory [3] to investigate parents’ attitudes towards public health officials and their COVID-19 vaccination intentions for their children. By examining the relative influence of environmental, behavioral, and personal factors per the TRD, the results of this study aim to provide direction as to how we might target future messaging to reduce parents’ vaccine hesitancy and slowing the spread of COVID-19. Our results show that child age and, especially, news media use behaviors and individual political attitudes, all uniquely shape parental responses to COVID-19.

COVID-19 has infected over 540 million people and caused the death of 6.32 million worldwide by the mid of June 2022 [4], where the United States has the greatest number. In the United States alone there have been approximately 86.5 million COVID-19 cases and over 1 million deaths (1.2% death rate) [5]. COVID-19 also influences people’s lives in other ways. For example, in November of 2011, 22% of U.S. parents reported that their child had missed at least four days of school due to COVID-19 [6]. COVID-19 infected individuals are also 35% more likely to suffer from anxiety disorders and nearly 40% more likely to experience depression or stress-related disorders [7].

Effective vaccines to battle COVID-19 were developed at an unprecedented speed [8]; By October 2021, there were 18 authorized COVID-19 vaccines in use around the world, with an additional 194 candidate vaccines in preclinical development and 126 in clinical stage of development [9]. In the United States, the U.S. Food and Drug Administration (FDA) issued the first vaccine emergency use authorization (EUA) available to people 16 years of age and older to Pfizer-BioNTech on 11 December 2020, [10]. Mass vaccination efforts have continued since then. In May 2020, adolescents from 12 to 15 years of age were authorized to receive a COVID-19 vaccine [11]. Next, federal officials approved Pfizer’s vaccine for children from 5 to 11 years old at the end of October 2021 [12]. On 17 June 2022, children under 6 months of age were approved to receive COVID-19 vaccines [13]. Vaccine hesitancy has slowed the impact of these efforts; although 76.4% of adults and 90.7% of elderly Americans were fully vaccinated [14], only about 28% of children aged 5 to 11 and 58% of those ages 12 to 17 were fully vaccinated by the middle of May, 2022 [15].

Vaccine hesitancy has been a public health issue for decades. Kumar et al. [16] described vaccine hesitancy as a continuum ranging from full acceptance to outright refusal. Behavioral responses to vaccine hesitancy are impacted by factors such as environmental interactions, agent/vaccine specific factors and host/parental specific factors. In addition, broader social-cultural factors contribute to vaccination decision-making [17]. To better understand these factors, this study investigated parental vaccine hesitancy against COVID-19 as guided by Bandura’s triadic reciprocal determinism (TRD). More specifically, this study modeled parents’ attitudes towards health officials and parents’ intentions to vaccinate their children against COVID-19 as related to the personal factors of partisan identification and age of the youngest child, the environmental factor of state of residence, and behavioral factor of news media use.

## 2. Literature Review

### 2.1. Triadic Reciprocal Determinism

The triadic reciprocal determinism model (TRD) is a conceptual framework found in social cognitive theory (SCT) that guides understanding of attitudes and behaviors. First articulated by Bandura and Locke [18,19], TRD illustrates the interplay among three determinants—personal factors (e.g., cognitive, affective, and biological events), environmental factors (e.g., physical surroundings, families and friends, as well as social influences) and behavior [20] in a reciprocal and dynamic manner [3,19]. TRD explains how our behavior impacts conditions in our environment, and subsequently how we react to the environmental feedback we receive [21]. It is important to note that the three components do not all necessarily hold the same weight in TRD [22].

The present study considers the role of personal, behavioral, and environmental factors on individual attitudes and behavioral intentions. In the following sections we will discuss why the personal factors of partisanship and age of youngest child, the behavioral factor of news media use, and the environmental factor of geographic location should be related to parent’s attitudes toward health officials as well as their intention to get children vaccinated. Guided by the TRD, we will discuss our key outcomes, attitudes, and behavioral intentions, followed by focal personal, behavioral, and environmental factor variables.

### 2.2. Attitudes towards Health Officials

Past social scientific research has demonstrated a longstanding link between attitudes and people’s health behaviors or behavioral intentions [23]. A recent study on COVID-19 vaccination in the United Kingdom found negative attitudes towards vaccination were major barriers to managing the pandemic in the long term [24]. Public attitudes towards health officials and healthcare providers also strongly influence health decision-making [25]; conversely, lack of trust in public health officials reduces utilization of health services [26]. Inconsistent risk messages from public health experts and elected officials may have led to greater transmission of the SARS-CoV-2 virus [1] and reduced vaccine uptake [27]. After the breakout of COVID-19, cues sent by polarized political elites in the United States influenced public attitudes and behaviors and hindered effective responses to the public health crisis [28]. Latkin et al. [29] found that people who have lower levels of trust in the Centers for Disease Control and Prevention (CDC) as a source of COVID-19 information were significantly less likely to get vaccinated. Another recent study by Viskupič et al. [30] reported a positive relationship between trust in physicians and the likelihood of COVID-19 vaccine uptake.

### 2.3. Intention to Vaccinate Children against COVID-19

The World Health Organization (WHO) recognizes vaccine hesitancy as a major threat to global health due to the resurgence of vaccine-preventable illnesses [31]. In the United States, the highest rates of COVID-19 vaccine acceptance of 72% were reported in early April 2020 [32]. By mid-October 2020, the number had dropped to 48% [33]. The SAGE working group on vaccine hesitancy stated that contextual factors of vaccine hesitancy included geographic area, media use, historical influences, culture, trust in healthcare professionals and systems, trust in policy makers, and trust in the pharmaceutical industry and the vaccine itself [34].

Trust in these various factors varies widely among parents. Previous studies found that parents reported trusting their children’s doctor for vaccine-safety information most often (76% endorsed a lot of trust), followed by other health care providers (26%), and government vaccine experts/officials (23%; [35]). Although many parents support vaccination, others (around 23%) believe that children get too many immunizations [36]. Parents in the U.S. are more vaccine hesitant in comparison to other countries. This study focuses on parents’ behavioral intent to vaccinate their children as a key dependent variable. Next, we will discuss our focal personal, behavioral, and environmental factor variables.

### 2.4. Partisanship

The COVID-19 pandemic has been wildly politicized, such that attitudes and behaviors regarding vaccination are strongly shaped by individuals’ political partisanship. For example, more Democrats (42%) as compared to Republicans (19%) indicated in October 2020 that the Coronavirus is a severe health threat [37]. Similarly, significantly more Democrats (80%) reported accepting a COVID-19 vaccine compared to Republicans (48%), as vaccines were in development in September 2020 [38]. Similarly, Democrats reported significantly higher COVID-19 vaccination intentions than others [39], while Republicans have indicated lower confidence in scientists and have been less likely to vaccinate self or children [40]. Bhanot and Hopkins [41] uncovered partisan differences on several COVID-related policies, but no difference in trust in medical experts. Rao et al. [42] found a significant correlation in polarized views along the science and political dimensions in Twitter. Politically moderate users were more aligned with pro-science views, while hardline users were more aligned with anti-science views. In sum, there is reason to expect the personal factor of individual partisanship to correlate with parents’ COVID attitudes and behaviors.

### 2.5. Age of Youngest Child

A child’s age is another personal factor that seems to guide parents’ attitudes and behaviors towards health officials and child vaccination [43]. Many parents believe the younger the child is, the riskier the vaccination becomes due to younger children’s under-developed immune system. These beliefs have led to a resurgence of vaccine hesitancy among parents of infants and young children in and out of the United States [44]. For example, the number of parents with children aged below two years refusing vaccination increased from 470 cases in 2013 to 1292 cases in 2014 in Malaysia [45]. Several other studies on COVID-19 vaccines found parents’ views on intention to vaccinate children varies. First, a survey panel conducted by Gallup [46] suggested that 55% of parents in the U.S. who have children under 12 would like to have their children vaccinated if available. However, Simonson et al. [47] found that parents with children younger than 12 were less likely to have their children vaccinated compared to parents with older children, which echoes with Szilagyi et al. [48]’s finding that the likelihood of child vaccination was greater among parents of older children.

Based on this past work on the personal factors of partisanship and children’s ages, we believe there will be a consistent pattern for our focal variables such that,

**H1:**  *The personal factors of partisanship (a) and the age of youngest child (b) will be related to attitudes towards health officials, where Republicans and parents of younger children will have more negative attitudes.*

**H2:**  *The personal factors of partisanship (a) and the age of youngest child (b) will be related to behavioral intention of vaccinating children, where Republicans and parents of younger children will have lower intentions to vaccine their children against COVID-19.*

### 2.6. News Media Use

Next, we discuss a focal behavioral factor of news media use. Given the partisan response to COVID-19 vaccinations discussed earlier, it is logical that partisan and non-partisan news media use could also influence COVID-19 attitudes and behaviors. Media messages have been shown to influence vaccination rates. Das et al. [49] analyzed online news on immunization and vaccines published in English in India during 2015 to 2020 and found that negative vaccine news took up a sizable portion of the publicized online news and influenced public vaccine sentiment and attitudes. Catalán-Matamoros and Peñafiel-Saiz [50] reviewed 24 communication studies exploring newspaper coverage of vaccines; 75% of the studies reviewed contained negative messages on vaccines and 83% identified a lack of accurate information. A specific study on pertussis immunization indicated that local newspaper campaigns brought media controversy on immunization and led to a reduction in MMR vaccine uptake [51].

More specifically, online news use presents unique challenges regarding information about COVID-19 and vaccination. The pandemic brought an unprecedented surge of polarization of political beliefs [52], resulting in online news organizations becoming increasingly partisan [53]. For example, conservative news media viewers were exposed more to vaccine conspiracy beliefs and reported lower intentions to vaccinate against COVID-19 [54]. Motta and Stecula [55] found that Fox News’ vaccine-related coverage was significantly more negative toward COVID-19 vaccines than other cable and mainstream sources. Additionally, exposure to Fox News in the opinion data was associated with higher levels of COVID vaccine hesitancy [55]. Intent to vaccinate was lower for Fox News users than CNN/MSNBC users [39]. Therefore, we predict that news media use will be a key behavioral factor related to attitudes toward health officials and child vaccination intentions. More specifically, we predict that conservative news media use will be related to more negative attitudes and lower behavioral intentions, whereas liberal and mainstream media use will be positively related to our focal variables.

**H3:**  *The behavioral factor of news media use, specifically (a) non-partisan mainstream news, (b) liberal news media, and (c) conservative news media, will be related to attitudes towards health officials, where use of conservative news media will be related to more negative attitudes.*

**H4:**  *The behavioral factor of news media use, specifically (a) non-partisan mainstream news, (b) liberal news media, and (c) conservative news media, will be related to behavioral intention of vaccinating children, where use of conservative news media will be related to lower intentions to vaccinate their child against COVID-19.*

### 2.7. Location

Lastly, we will investigate an environmental factor of geographic location. Since its outbreak, COVID-19 has impacted countries and regions differently. Li et al. [56] selected six provinces based on geographic locations in China and found that the proportion of intention to receive COVID-19 vaccination from each province varies. Our study focuses on four U.S. states with differing levels of partisan diversity. TRD posits that a person’s environment will influence their attitudes and behaviors. Indeed, past research has showed that regional cultures, like cultural tightness–looseness or political affiliations, shaped how people react to the crisis like COVID-19 [57]. Jiang et al. [58] found that Democratic- and Republican-leaning states differed considerably in topics of conversations pertaining to COVD-19. They found that COVID-19 conversations were largely political in nature and polarized along partisan lines. Chen and Karim [59] found a significant trend in COVID-19 death rates between Democratic and Republican counties, which were influenced by the politically polarized response to the pandemic. They found that Democratic counties showed higher death rates at the outset of the COVID-19 pandemic in 2020 and plateaued throughout the year. Republican counties trended positively in their death rates over the year and by the end of 2020 showed significantly higher death rates than Democratic counties. Chen and Karim [59] argued these geographic results illustrate the environmental consequences of the polarized response to the pandemic. In another illustration of this trend, Morning Consult [32] revealed a regional difference in willingness to receive COVID-19 vaccination between the northeast at 38% and the west at 49%. Rao et al. [42] also indicated that anti-science conservatives in the United States tended to tweet from the southern and northwestern states, while anti-science moderates tended to tweet from the western states.

Past research also suggested a difference among parent’s attitudes and behaviors toward COVID-19 vaccine. For example, in August 2020, parents were split about whether schools should require COVID-19 vaccination, with 54% opposed to a mandate [60]. These attitudes largely fell in line with parental behaviors, with 75% of vaccinated parents preferring a vaccine mandate and 83% of unvaccinated parents opposed such a mandate [60]. These results illustrate systematic geographic trends where states with the greatest vaccine uptake trend match partisan preferences. In fact, over 20 of the top 25 states in child and adult vaccine uptake are states that voted for Joe Biden in the 2020 election [61]. State-level school policies also influence vaccine uptake. Sixteen U.S. states with Republican state-level leadership have passed measures that ban school vaccine mandates [62]; in contrast, Democratic-led states and the federal Biden administration encouraged mandatory vaccine policies [63].

TRD posits that environmental factors such as these will uniquely influence attitudes and behaviors, alongside personal and behavioral factors. We expect people’s individual COVID-19 responses will vary by state, which have unique political leadership and differing political communities. Based on these previous findings and theoretical guidance relative to environmental factors under TRD, we propose the following hypotheses.

**H5:**  *The environmental factor of geographic location will be related to attitudes towards health officials, where people from more politically conservative states (Texas, Ohio) will hold more negative attitudes than those in more politically liberal states (New York, Georgia).*

**H6:**  *The environmental factor of geographic location will be related to behavioral intention of vaccinating children, where people from more politically conservative states (Texas, Ohio) will have lower intentions to vaccinate their children against COVID-19 than people in more politically liberal states (New York, Georgia).*

## 3. Methods

### 3.1. Participants and Procedures

After receiving approval from the university institutional review board, an anonymous online survey was administered in March 2021 through YouGov. The sample included 800 sample-matched parents of school-aged children from 4 states (200 each from Ohio, New York, Georgia, and Texas). These four states were chosen by the researchers to represent a variety of geographic areas, partisan populations, responses to the pandemic (early lockdowns and school closures, late lockdowns and school closures, no lockdowns or closures, early/late releases of lockdowns), and severity of the state’s COVID-19 outbreak. YouGov’s sample matching procedure initially over-samples participants, then generates a final sample that matches national population characteristics using a sample frame from large, high quality probably samples. This procedure has been found to exhibit little to no selection bias [64,65]. We excluded 55 respondents who indicated that they had already had their children vaccinated. After excluding responses with missing data, a total of 714 responses were included in our sample.

### 3.2. Measures

Independent variables included age group for the youngest child, media use, partisanship, and state of residence, and demographic variables, such as gender, race, highest education level, and marital status. Dependent variables were intention to vaccinate children and attitude towards health officials.

#### 3.2.1. Age Group for the Youngest Child

Participants reported how many children they have and the age of each child. We then recoded the youngest child from every family into three groups: 0 to 4 years old, 5 to 11 years old, and 12 years old and above (see Table 1). Age group categories were based on the COVID-19 vaccine availability and ongoing clinical trials. At the time of our March 2021 data collection, vaccines were only available under an EUA for people 16 years and older. As noted earlier, subsequent authorizations were granted after these data were collected for children ages 12–15, then ages 5–11, and finally under 5 years of age.

#### 3.2.2. News Media Use

Neutral, conservative, and liberal news media use were measured with three items each where participants self-reported their frequency of viewing news from a national news organization, website of a major national news organization, and website of an online news organization that is frequently characterized as not favoring a particular political party, favoring liberal positions or Democratic candidates, and favoring conservative positions or Republican candidates [66]. Respondents reported how often they use news media with six-point Likert scale (1 = never, 6 = several times a day). Cronbach’s alpha coefficients indicated the scale is reliable: conservative media use (M = 2.28, SD = 0.07, α= 0.86), neutral media use (M = 2.73, SD = 0.07, α= 0.81), and liberal media use (M = 2.43, SD = 0.07, α= 0.84).

#### 3.2.3. Partisanship

Seven choices (strong Democrat, not very strong Democrat, lean Democrat, Independent, lean Republican, not very strong Republican, strong Republican), coded from 1 (strong Democrat) to 7 (strong Republican), were used to quantify partisanship among participants (M = 3.80, SD = 0.11). For individuals answering “not sure” for this measure, their three-point party identification results and their presidential election voting results in 2016 and 2020 were imputed. Specifically, individuals answering “Independent” in the three-point party ID question were recoded as “Independent” in partisanship, while others answering “not sure” were recoded based on voting results in 2016 and 2020. Individuals who indicated consistent voting preference for Democratic candidates were recoded as “lean Democrat”, while individuals who consistently voted for a Republican candidate were recoded as “lean Republican.” The remaining individuals who only voted for candidates not from two major parties or who did not vote consistently for a major party at both times were discarded as missing data.

#### 3.2.4. State of Residence

All the participants were recruited from four states according to their residencies, including New York, Ohio, Georgia, and Texas (see Table 1). States were dummy coded with New York used as the reference group.

#### 3.2.5. Attitude towards Health Officials

Attitude towards health officials were measured by asking participants to rate three items related to health officials including (Dr. Tony Fauci [prominent U.S. public health official who was a member of the White House Coronavirus Task Force], doctors and nurses, as well as public health officials) between 0 degrees and 100, respectively (M = 62.76, SD = 1.34, α = 0.83). For example, 88 degrees means they felt favorable and “warm” toward the person or the group, whereas 23 degrees meant that they didn’t feel favorable or warm toward the person or the group.

#### 3.2.6. Intention to Vaccinate Child

Intention to vaccinate children was measured with two binary yes/no questions asking if people plan to get their children vaccinated with the COVID-19 vaccine in the future and if they would be willing to get their children vaccinated if the COVID-19 vaccine were available in their doctor’s office. As long as people answered “yes” to one of the two questions, their intention to vaccinated children was coded as “yes” (see Table 1).

#### 3.2.7. Control Variables

Standard demographic information was collected including gender, race, highest education level, and marital status. See Table 1 for descriptive details for control variables.

### 3.3. Analysis Plan

All statistical analyses were conducted in R. We employed ordinary least-squares linear regression and logistic regression to model the dependent variables. Analyses were run using weights supplied by YouGov.

## 4. Results

We conducted a multiple linear regression analysis to identify the association between age group of the youngest child, partisanship, location, news use, and attitude towards health officials (see Table 2). The variance inflation factor (VIF) for each independent variable is also presented in Table 2 to assess the possibility of collinearity. None of them is larger than 5, which is the signal for concern.

The regression results suggested that age group of youngest child, partisanship, and media use were significantly related to attitude towards health officials. Specifically, compared to those parents with the youngest child aged 12 years old or above, parents with the youngest child aged 0 to 4 years old had more negative attitudes toward health officials (B = −6.53, SE = 2.10, *p* < 0.01), supporting H1a. With one unit more towards Republicans in partisanship, the predicted attitude towards health officials decreased 3.60 degrees (B = −3.60, SE = 46, *p* < 0.001), supporting H1b. Moreover, liberal media usage (B = 2.97, SE = 1.02, *p* < 0.01) and neutral media usage (B = 5.78, SE = 0.94, *p* < 0.001) were positively related to more positive attitudes toward health officials, supporting H3a and H3b. Conservative media usage (B = −6.68, SE = 0.80, *p* < 0.001) was negatively related to attitudes toward health officials, supporting H3c. We found no relationship between geographic location in our 4 selected states and attitudes towards health officials (see Table 2). Therefore, we find no support for H5. The overall model predicted a substantive part of the variation of attitudes toward health officials (Adj. R^2^ = 0.37).

Additionally, we conducted a post-hoc analysis to compare the relative impacts of the factors on attitudes toward health officials. We applied Cumming’s [67] approach to examine the standardized coefficient of each variable within the linear regression model and the overlap of confidence intervals of significant factors. Cumming [67] showed that if the overlap between confidence intervals is less than 50%, a focal variable has a significantly stronger effect than another variable. In our results, the confidence intervals between liberal media use (CI 0.05, 0.26) and neutral media use (CI 0.21, 0.41), conservative media (CI −0.42, −0.26), and partisanship (CI −0.38, −0.22) are less than 50%, so we conclude that partisanship, conservative media use, and neutral media use have significantly stronger relationships with health official attitudes than liberal media use. Similarly, partisanship, conservative media use, and neutral media use have significantly stronger relationships than age group 0–4 (CI −0.18, −0.04). In this case, partisanship and media use are crucial factors to predict attitudes toward health officials. Specifically, results show that Republican partisanship and conservative media use hold the strongest negative association with attitudes toward health officials.

We used a multiple logistic regression model to examine the relationship between age group of the youngest child, partisanship, location, media use, and intention for children vaccination against COVID-19 (see Table 3).

The results from this model suggested that age group of the youngest child, media use, and partisanship were significantly associated with intention of COVID-19 vaccination for children. Specifically, compared to those who have the youngest child aged 12 and above, parents with youngest child aged between 0–4 were less likely to have the intention for their children to be vaccinated (OR = 0.41, 95% CI 0.22–0.77), supporting H2a. Individuals who were closer to Republicans in partisanship had lower intentions for their children to be vaccinated (OR = 0.83, 95% CI 0.71–0.97), supporting H2b. Individuals with more conservative media usage were less likely to have the intention for their children to be vaccinated (OR = 0.77, 95% CI 0.60–0.98), supporting H4c. Individuals with more liberal media usage were more likely to have the intention for their children to be vaccinated (OR = 1.42, 95% CI 1.07–1.88), supporting H4a. We found no significant relationship between neutral news media use or state of residence (see Table 3). Therefore, we do not support H4c or H6.

## 5. Discussion

In this study, we attempted to shed light on the influence of the personal factors (youngest child’s age and partisanship), an environmental factor (the state where they reside) and a behavioral factor (media use) on parents’ attitudes toward health officials, as well as their intention to vaccinate their child, as guided by the triadic reciprocal determinism model from social cognitive theory. Overall, our results underline the importance of partisanship in forming attitudes toward health officials and intention to get children vaccinated. Again, illustrating the politicization of the pandemic, we found that partisan news use was related to parents’ attitudes toward health officials and their behavioral intentions regarding vaccinating their children against COVID-19. We found that the children’s age in the household influences parents’ attitude toward health officials and their attitudes toward getting children vaccinated. In summary, parents who are Republican partisans, conservative news users, and have children in the youngest age group held significantly more negative attitudes towards health officials and were less likely to intend to vaccinate their children. In contrast, Democratic partisans and liberal news users held significantly more positive attitudes towards health officials and were more likely to intend to vaccinate their children. We found a similar pattern for non-partisan news users as liberal news users, but non-partisan news users only showed statistically significant differences in their positive attitudes towards health officials. We found no differences in our dependent variables based on our participants’ state of residence, which served as our environmental factor variable.

This study makes several contributions to the existing literature that focuses on the TRD in social cognitive theory as well to the COVID-19 vaccine literature. First, we were able to utilize the TRD model by applying personal, behavioral, and environmental factors to the study of a volatile and emergent health concern (vaccinating children against COVID-19). We extend research at the intersection of political and health communication by demonstrating partisanship and political news media behaviors uniquely shape health attitudes and behavioral intentions. Second, our study examines parents’ attitudes towards health officials and intentions of vaccinating their child. Our findings could provide practical guidance for health officials to focus promotion of the COVID-19 vaccines among the younger groups of children, especially with the group aged 0 to 4 years old, the final age group to gain access to the COVID-19. While TRD proves a useful framework for modeling attitudes and behaviors, we found no relationship between our environmental variable and our outcome variable. This contributes to our understanding of social science by illustrating that it is not necessarily one’s neighbors and local political entities that influence attitudes and behaviors, but instead one’s preferred political party. That is, as political polarization continues to increase over time and the communication environment becomes further fragmented, partisan identities seem to dominate attitudes and behaviors relating to an increasing number of politicized issues.

These results have implications for public health messaging around politicized health issues. Specifically, we believe it is worthwhile to prioritize public health messaging around child vaccination toward more conservative Republican parents. It may also be important to design and fund research focused on messaging that can help break through the polarized partisan skepticism surrounding vaccines coming from Republican leaders and members. Some early research suggests that messages containing universal orientation may transcend partisan identity. For example, Oliver et al. [68] found that uplifting videos focusing on patriotic American values, such as the flag and military, moderated partisan differences on mask-wearing during the COVID-19 pandemic. Similarly, source credibility theory suggests that corrections from Republicans speaking against their partisan interests can persuade respondents to acknowledge and agree with the scientific consensus on anthropogenic climate change [69] may be also effective in achieving this goal for vaccination.

Our post-hoc analysis showed that partisanship, conservative media use, and neutral media use held the strongest associations with attitudes towards health officials. In fact, our research provides novel understanding that media use is a crucial factor in people’s attitude towards health officials. Pairing our findings that Republican partisans and conservative media use are strongly associated with negative attitudes towards health officials and child vaccination, these findings indicate future research should focus on tailored messaging targeting conservative viewers. These political factors were stronger than the environmental factor of geography or age of one’s youngest child. We also found distinct differences between liberal and conservative media and individual partisan identity. More study is needed in this area, as our study suggests that partisanship is a primary barrier towards universal trust in public health officials and vaccine acceptance for children.

Like all studies, our research also has limitations. First, the four states where the survey was conducted (New York, Georgia, Texas, and Ohio) were chosen by the authors according to the voting results for 2020 presidential election and geographical locations distinguished by the South and North at a state level. However, the nuances among individual participants’ geographic environment may vary widely, which is hard to generalize by state level. Future studies should extend the measurement for the environmental factors in the TRD model beyond the state level only. For instance, future research focusing on smaller contextual units, such as the municipality, county, or neighborhood (e.g., zip code) may lead to different findings relative to environmental factors. Zip codes based on individuals’ home address might better capture the nuances of community-contextual environment [70]. Another way of defining the environmental factor is not measuring the geographic community, but examining the network community of the participants. In addition, research has found other individual factors, such as psychological impact (stress and depression), are important predictors of people’s behaviors during COVID-19 [71]. Furthermore, our study relied on self-reported news media use, which can be vulnerable to social desirability bias [72]. A future study might collect behavioral trace data that pairs with health survey data or even medical records to reduce measurement error related to the desirability bias of pro-social news use or health behavior.

## 6. Conclusions

Our study’s findings converge with a growing body of important research at the intersection of political and health communication that shows how partisanship is a growing concern for public health practitioners, especially as the COVID-19 mitigation strategies become increasingly polarized [73,74]. We add to this political communication work by showing how the partisan variables fit into the TRD framework. Moreover, our work shows the stronger negative attitudes and vaccination intention from parents of younger children. This study contributes to the growing literature that provides evidence for health officials, policymakers, and pediatric medical professionals to better understand the opportunities and challenges in the uptake of the COVID-19 vaccination to children.

## Figures and Tables

**Table 1:** Descriptive information about the sample (N = 714).

	Unweighted N	Weighted Mean/Percentage	95% CI
Age group for the youngest child			
12 and above	240	32.92	28.10–37.74
5–11	302	42.68	37.42–47.95
0–4	172	24.40	19.81–28.98
Gender			
Male	282	42.61	37.31–47.92
Female	432	57.39	52.08–62.69
Race			
White	438	47.39	42.21–52.57
Black	101	16.28	12.37–20.18
Other	175	36.33	30.77–41.89
Education			
No high school	31	11.22	6.42–16.03
High school graduate	159	26.27	21.40–31.14
College experience	524	62.51	56.93–68.08
Marital status			
Married	471	66.07	61.09–71.04
Separated	20	3.28	1.29–5.26
Divorced	68	9.73	6.46–12.99
Widowed	16	2.30	0.78–3.83
Never married	91	12.39	9.02–15.75
Domestic/civil partners	48	6.24	3.87–8.60
Partisanship (R)	714	3.77	3.55–3.99
State of residence			
New York	177	27.58	22.95–32.22
Georgia	174	14.95	12.02–17.88
Ohio	189	15.87	13.06–18.68
Texas	174	41.59	36.08–47.11
Media use			
Liberal	714	2.44	2.30–2.57
Neutral	714	2.73	2.59–2.88
Conservative	714	2.28	2.15–2.42
Child vaccination intention			
Yes	464	60.25	54.98–65.52
No	250	39.75	34.48–45.02
Attitude towards health officials	714	62.76	60.14–65.39

**Table 2:** Results for the OLS regression model.

	Attitudes toward Health Officials				
	B	β	SE	95% Standardized CI	VIF
Age group for the youngest child					1.15
12 and above	reference	reference			
5–11	−2.92	−0.06	1.02	[−0.13, 0.01]	
0–4	−6.53 **	−0.11 **	2.10	[−0.18, −0.04]	
Partisanship (R)	−3.60 ***	−0.30 ***	0.46	[−0.38, −0.22]	1.66
Location					1.48
New York	reference	reference			
Georgia	−2.16	−0.04	2.53	[−0.12, 0.05]	
Ohio	−1.75	−0.03	2.50	[−0.12, 0.06]	
Texas	1.88	0.03	1.98	[−0.03, 0.10]	
Media Use					
Liberal	2.97 **	0.16 **	1.02	[0.05, 0.26]	3.05
Neutral	5.78 ***	0.31 ***	0.94	[0.21, 0.41]	2.75
Conservative	−6.68 ***	−0.34 ***	0.80	[−0.42, −0.26]	1.88
Additional controls					
Gender					1.10
Male	reference	reference			
Female	1.88	0.04	1.60	[−0.03, 0.10]	
Race					1.82
White	reference	reference			
Black	−4.22	−0.06	2.48	[−0.13, 0.01]	
Other	1.38	0.02	1.96	[−0.04, 0.09]	
Education					1.23
No high school	reference	reference			
High school graduates	−1.67	−0.03	2.87	[−0.12, 0.07]	
College experience	3.20	0.06	2.64	[−0.04, 0.15]	
Marital status					1.55
Married	reference	reference			
Separated	−6.63	−0.04	4.39	[−0.10, 0.01]	
Divorced	−4.56	−0.05	2.65	[−0.12, 0.01]	
Widowed	9.01	0.05	5.33	[−0.01, 0.12]	
Never married	0.55	0.01	2.58	[−0.06, 0.08]	
Domestic/civil partners	0.86	0.01	3.24	[−0.06, 0.07]	
F	22.69 (19, 694)				
Adj. R^2^	0.37				

*** *p* < 0.001, ** *p* < 0.01.

**Table 3:** Results for the multiple logistic regression analysis.

	Child Vaccination Intention	
	OR	95% CI
Age group for the youngest child		
12 and above	reference	
5–11	0.97	0.51–1.75
0–4	0.41 *	0.22–0.77
Partisanship (R)	0.83 *	0.71–0.97
State of residence		
New York	reference	
Georgia	0.71	0.36–1.40
Ohio	0.88	0.48–1.60
Texas	0.55	0.29–1.06
Media Use		
Liberal	1.42 *	1.07–1.87
Neutral	1.18	0.88–1.59
Conservative	0.77 *	0.60–0.98
Additional controls		
Gender		
Male	reference	
Female	1.02	0.60–1.73
Race		
White	reference	
Black	0.33 **	0.15–0.74
Other	1.21	0.63–2.31
Education		
No high school	reference	
High school graduates	1.02	0.31–3.30
College experience	1.73	0.61–4.87
Marital status		
Married	reference	
Separated	0.71	0.25–2.05
Divorced	0.47	0.19–1.14
Widowed	14.91 **	2.54–87.60
Never married	0.39 *	0.19–0.80
Domestic/civil partners	0.92	0.32–2.62

** *p* < 0.01, * *p* < 0.05.

## Data Availability

The data presented in this study are available on request from the corresponding author. The data are not publicly available due to privacy concerns.

## References

[1] Panchalingam T., Shi Y. (2022). Parental refusal and hesitancy of vaccinating children against COVID-19: Findings from a nationally representative sample of parents in the U.S. Prev. Med..

[2] Williams S.E. (2014). What Are the Factors That Contribute to Parental Vaccine-Hesitancy and What Can We Do about It?. Hum. Vaccines Immunother..

[3] Bandura A. (2001). Social Cognitive Theory: An Agentic Perspective. Annu. Rev. Psychol..

[4] WHO WHO Coronavirus (COVID-19) Dashboard. https://covid19.who.int/.

[5] JHCRC (2022). Mortality Analyses. John Hopkins Coronavirus Resources Center. https://coronavirus.jhu.edu/data/mortality.

[6] Blad E. (2022). Chronic Absenteeism Spiked during COVID. Here’s What Schools Can Do about It. Education Week. https://www.edweek.org/leadership/chronic-absenteeism-spiked-during-covid-heres-what-schools-can-do-about-it/2022/04#:~:text=In%20a%20November%202021%20survey,least%20four%20days%20of%20school.

[7] Sauerwein K. COVID-19 Survivors Face Increased Mental Health Risks up to a Year Later. Washington University School of Medicine in St. Louis. https://medicine.wustl.edu/news/covid-19-survivors-face-increased-mental-health-risks-up-to-a-year-later/#:~:text=Compared%20with%20those%20in%20the,can%20affect%20behavior%20and%20emotions.

[8] Ndwandwe D., Wiysonge C.S. (2021). COVID-19 Vaccines. Curr. Opin. Immunol..

[9] WHO COVID-19 Vaccine Tracker and Landscape. https://covid19.who.int/.

[10] FDA FDA Takes Key Action in Fight against COVID-19 by Issuing Emergency Use Authorization for First COVID-19 Vaccine. https://www.fda.gov/news-events/press-announcements/fda-takes-key-action-fight-against-covid-19-issuing-emergency-use-authorization-first-covid-19.

[11] FDA Coronavirus (COVID-19) Update: FDA Authorizes Pfizer-BioNTech COVID-19 Vaccine for Emergency Use in Adolescents in Another Important Action in Fight Against Pandemic. https://www.fda.gov/news-events/press-announcements/coronavirus-covid-19-update-fda-authorizes-pfizer-biontech-covid-19-vaccine-emergency-use.

[12] FDA FDA Authorizes Pfizer-BioNTech COVID-19 Vaccine for Emergency Use in Children 5 through 11 Years of Age. https://www.fda.gov/news-events/press-announcements/fda-authorizes-pfizer-biontech-covid-19-vaccine-emergency-use-children-5-through-11-years-age.

[13] FDA Coronavirus (COVID-19) Update: FDA Authorizes Moderna and Pfizer–BioNTech COVID-19 Vaccines for Children down to 6 Months of Age. https://www.fda.gov/news-events/press-announcements/coronavirus-covid-19-update-fda-authorizes-moderna-and-pfizer-biontech-covid-19-vaccines-children.

[14] CDC COVID-19 Vaccinations in the United States. https://covid.cdc.gov/covid-data-tracker/#vaccinations_vacc-total-admin-rate-total.

[15] CDC COVID-19 Vaccinations in the United States, Jurisdiction. https://data.cdc.gov/Vaccinations/COVID-19-Vaccinations-in-the-United-States-Jurisdi/unsk-b7fc.

[16] Kumar D., Chandra R., Mathur M., Samdariya S., Kapoor N. (2016). Vaccine Hesitancy: Understanding Better to Address Better. Isr. J. Health Policy Res..

[17] Stefanoff P., Løvlie A.L., Elstrøm P., Macdonald E.A. (2020). Registrerte meldingspliktige smittsomme sykdommer under COVID-19-responsen. Tidsskr. Nor. Legeforening.

[18] Bandura A. (1978). The Self System in Reciprocal Determinism. Am. Psychol..

[19] Locke E.A., Bandura A. (1987). Social Foundations of Thought and Action: A Social-Cognitive View. Acad. Manag. Rev..

[20] Wood R., Bandura A. (1989). Social Cognitive Theory of Organizational Management. Acad. Manag. Rev..

[21] Rice K. (2007). Knowing Me, Knowing You: An Integrated SocioPsychology Guide to Personal Fulfillment & Better Relationships.

[22] Lo Schiavo M., Prinari B., Saito I., Shoji K., Benight C.C. (2019). A Dynamical Systems Approach to Triadic Reciprocal Determinism of Social Cognitive Theory. Math. Comput. Simul..

[23] Baiocchi-Wagner E.A., Talley A.E. (2013). The Role of Family Communication in Individual Health Attitudes and Behaviors Concerning Diet and Physical Activity. Health Commun..

[24] Paul E., Steptoe A., Fancourt D. (2021). Attitudes towards Vaccines and Intention to Vaccinate against COVID-19: Implications for Public Health Communications. Lancet Reg. Health Eur..

[25] Bavel J.J.V., Baicker K., Boggio P.S., Capraro V., Cichocka A., Cikara M., Crockett M.J., Crum A.J., Douglas K.M., Druckman J.N. (2020). Using Social and Behavioural Science to Support COVID-19 Pandemic Response. Nat. Hum. Behav..

[26] Alsan M., Wanamaker M. (2018). Tuskegee and the Health of Black Men. Q. J. Econ..

[27] Thunstrom L., Ashworth M., Finnoff D., Newbold S. (2020). Hesitancy Towards a COVID-19 Vaccine and Prospects for Herd Immunity. SSRN J..

[28] Green J., Edgerton J., Naftel D., Shoub K., Cranmer S.J. (2020). Elusive Consensus: Polarization in Elite Communication on the COVID-19 Pandemic. Sci. Adv..

[29] Latkin C.A., Dayton L., Yi G., Konstantopoulos A., Boodram B. (2021). Trust in a COVID-19 Vaccine in the U.S.: A Social-Ecological Perspective. Soc. Sci. Med..

[30] Viskupič F., Wiltse D.L., Meyer B.A. (2022). Trust in Physicians and Trust in Government Predict COVID-19 Vaccine Uptake. Soc. Sci. Q..

[31] WHO Ten Threats to Global Health in 2019. https://www.who.int/news-room/spotlight/ten-threats-to-global-health-in-2019.

[32] Morning Consult National Tracking Poll #200409. https://assets.morningconsult.com/wp-uploads/2020/04/200409_crosstabs_CORONAVIRUS_CONTENT_Adults_v4_JB-1.pdf.

[33] Galvin G. 48% of U.S. Adults Now Say They’d Get a COVID-19 Vaccine, a New Low. https://morningconsult.com/2020/10/12/48-of-u-s-adults-now-say-theyd-get-a-covid-19-vaccine-a-new-low/.

[34] MacDonald N.E. (2015). Vaccine Hesitancy: Definition, Scope and Determinants. Vaccine.

[35] Freed G.L., Clark S.J., Butchart A.T., Singer D.C., Davis M.M. (2011). Sources and Perceived Credibility of Vaccine-Safety Information for Parents. Pediatrics.

[36] (2002). News in Brief. Expert Rev. Vaccines.

[37] Galvin G., Bracken M. The Return to Normal: Views on the Pandemic. https://morningconsult.com/views-on-the-pandemic/.

[38] Jaimungal C. Concerns over Fast-Tracked COVID-19 Vaccine Has Americans Unsure about Vaccination. https://today.yougov.com/topics/health/articles-reports/2020/09/15/concerns-over-fast-tracked-covid-19-vaccine-has-am.

[39] Ruiz J.B., Bell R.A. (2021). Predictors of Intention to Vaccinate against COVID-19: Results of a Nationwide Survey. Vaccine.

[40] Viswanath K., Bekalu M., Dhawan D., Pinnamaneni R., Lang J., McLoud R. (2021). Individual and Social Determinants of COVID-19 Vaccine Uptake. BMC Public Health.

[41] Bhanot S.P., Hopkins D.J. (2020). Partisan Polarization and Resistance to Elite Messages: Results from Survey Experiments on Social Distancing. J. Behav. Public Adm..

[42] Rao A., Morstatter F., Hu M., Chen E., Burghardt K., Ferrara E., Lerman K. (2021). Political Partisanship and Antiscience Attitudes in Online Discussions About COVID-19: Twitter Content Analysis. J. Med. Internet Res..

[43] Mendel-Van Alstyne J.A., Nowak G.J., Aikin A.L. (2018). What is ‘Confidence’ and What Could Affect It?: A Qualitative Study of Mothers Who Are Hesitant about Vaccines. Vaccine.

[44] Wong L.P., Wong P.F., AbuBakar S. (2020). Vaccine Hesitancy and the Resurgence of Vaccine Preventable Diseases: The Way Forward for Malaysia, a Southeast Asian Country. Hum. Vaccines Immunother..

[45] Mohd Azizi F.S., Kew Y., Moy F.M. (2017). Vaccine Hesitancy among Parents in a Multi-Ethnic Country, Malaysia. Vaccine.

[46] Brenan M. In U.S., 55% Would Get COVID-19 Vaccine for Young Child. https://news.gallup.com/poll/354998/covid-vaccine-young-child.aspx.

[47] Simonson M.D., Chwe H., Lazer D., Ognyanova K., Baum M., Perlis R.H., Druckman J., Santillana M., Green J., Uslu A. (2021). The COVID States Project #49: Vaccinating America’s Youth.

[48] Szilagyi P.G., Thomas K., Shah M.D., Vizueta N., Cui Y., Vangala S., Kapteyn A. (2021). National Trends in the US Public’s Likelihood of Getting a COVID-19 Vaccine—April 1 to December 8, 2020. JAMA.

[49] Das M.K., Singh D., Sharma S. (2021). Media News on Vaccines and Vaccination: The Content Profile, Sentiment and Trend of the Online Mass Media during 2015–2020 in India. Clin. Epidemiol. Glob. Health.

[50] Catalán-Matamoros D., Peñafiel-Saiz C. (2019). Medios y Desconfianza En Vacunas: Un Análisis de Contenido En Titulares de Prensa. Rev. Lat. Comun. Soc..

[51] Mason B.W. (2000). Impact of a Local Newspaper Campaign on the Uptake of the Measles Mumps and Rubella Vaccine. J. Epidemiol. Community Health.

[52] Iyengar S., Lelkes Y., Levendusky M., Malhotra N., Westwood S.J. (2019). The Origins and Consequences of Affective Polarization in the United States. Annu. Rev. Polit. Sci..

[53] Garimella K., Smith T., Weiss R., West R. (2021). Political Polarization in Online News Consumption. arXiv.

[54] Van Green T., Tyson A. 5 Facts about Partisan Reactions to COVID-19 in the U.S. https://www.pewresearch.org/fact-tank/2020/04/02/5-facts-about-partisan-reactions-to-covid-19-in-the-u-s/.

[55] Motta M., Stecula D. (2021). Quantifying the Effect of Wakefield et al. (1998) on Skepticism about MMR Vaccine Safety in the U.S. PLoS ONE.

[56] Li L., Jing R., Guo J., Song Y., Geng S., Wang J., Zhang H., Lai X., Lyu Y., Feng H. (2021). The Associations of Geographic Location and Perceived Risk of Infection with the Intentions to Get Vaccinated against COVID-19 in China. Expert Rev. Vaccines.

[57] Lu J.G., Jin P., English A.S. (2021). Collectivism Predicts Mask Use during COVID-19. Proc. Natl. Acad. Sci. USA.

[58] Jiang J., Chen E., Yan S., Lerman K., Ferrara E. (2020). Political Polarization Drives Online Conversations about COVID-19 in the United States. Hum. Behav. Emerg. Technol..

[59] Chen H.-F., Karim S.A. (2022). Relationship between Political Partisanship and COVID-19 Deaths: Future Implications for Public Health. J. Public Health.

[60] Palosky C. Most Parents Don’t Want Their Schools to Require COVID-19 Vaccination, But Most Favor Requiring Masks for Unvaccinated Children and Staff. https://www.kff.org/coronavirus-covid-19/press-release/most-parents-dont-want-their-schools-to-require-covid-19-vaccination-but-most-favor-requiring-masks-for-unvaccinated-children-and-staff/.

[61] Enten H. How Children Are Exacerbating the Vaccine Divide between Blue and Red States. https://www.cnn.com/2021/05/30/politics/vaccinations-covid-children-analysis/index.html.

[62] Perez J. Red States Resist School Vaccine Mandates. https://www.politico.com/newsletters/weekly-education/2021/10/04/red-states-resist-school-vaccine-mandates-797995.

[63] Camera L. School Vaccine Mandates: Here They Come. https://www.usnews.com/news/national-news/articles/2021-08-31/school-vaccine-mandates-here-they-come.

[64] Ansolabehere S., Rivers D. (2013). Cooperative Survey Research. Annu. Rev. Political Sci..

[65] Ansolabehere S., Schaffner B. (2014). Does Survey Mode Still Matter? Findings from a 2010 Multi-Mode Comparison. Political Anal..

[66] Hmielowski J., Hutchens M., Beam M. (2020). Asymmetry of Partisan Media Effects?: Examining the Reinforcing Process of Conservative and Liberal Media with Political Beliefs. Political Commun..

[67] Cumming G. (2019). Inference by eye: Reading the overlap of independent confidence intervals. Stat. Med..

[68] Oliver M.B., Zhang B., Berndt M., Drivas M. (2022). Inspired to Mask up: The Effect of Uplifting Media Messages on Attitudes about Wearing Face Masks among Democrats and Republicans. Psychol. Pop. Media.

[69] Benegal S.D., Scruggs L.A. (2018). Correcting Misinformation about Climate Change: The Impact of Partisanship in an Experimental Setting. Clim. Chang..

[70] McLeod J.M. (2001). Steven Chaffee and the Future of Political Communication Research. Political Commun..

[71] Maliki I., Elmsellem H., Hafez B., El-Moussaoui A., Kachmar M.R., Ouahbi A. (2021). The psychological properties of the Arabic BDI-II and the psychological state of the general Moroccan population during the mandatory quarantine due to the COVID-19 pandemic. Casp. J. Environ. Sci..

[72] Prior M. (2009). The Immensely Inflated News Audience: Assessing Bias in Self-Reported News Exposure. Public Opin. Q..

[73] Clinton J., Cohen J., Lapinski J., Trussler M. (2021). Partisan Pandemic: How Partisanship and Public Health Concerns Affect Individuals’ Social Mobility during COVID-19. Sci. Adv..

[74] Gadarian S.K., Goodman S.W., Pepinsky T.B. (2021). Partisanship, Health Behavior, and Policy Attitudes in the Early Stages of the COVID-19 Pandemic. PLoS ONE.

